# A 4-fJ/Spike Artificial Neuron in 65 nm CMOS Technology

**DOI:** 10.3389/fnins.2017.00123

**Published:** 2017-03-15

**Authors:** Ilias Sourikopoulos, Sara Hedayat, Christophe Loyez, François Danneville, Virginie Hoel, Eric Mercier, Alain Cappy

**Affiliations:** ^1^Centre National de la Recherche Scientifique, Université Lille, USR 3380 - IRCICALille, France; ^2^Centre National de la Recherche Scientifique, Université Lille, ISEN, Université Valenciennes, UMR 8520 - IEMNLille, France; ^3^Université Grenoble Alpes, GrenobleGrenoble, France; ^4^CEA, LETI, MINATEC CampusGrenoble, France

**Keywords:** artificial neuron, Morris-Lecar neuron, CMOS, subthreshold, analog VLSI, spiking neural network

## Abstract

As Moore's law reaches its end, traditional computing technology based on the Von Neumann architecture is facing fundamental limits. Among them is poor energy efficiency. This situation motivates the investigation of different processing information paradigms, such as the use of spiking neural networks (SNNs), which also introduce cognitive characteristics. As applications at very high scale are addressed, the energy dissipation needs to be minimized. This effort starts from the neuron cell. In this context, this paper presents the design of an original artificial neuron, in standard 65 nm CMOS technology with optimized energy efficiency. The neuron circuit response is designed as an approximation of the Morris-Lecar theoretical model. In order to implement the non-linear gating variables, which control the ionic channel currents, transistors operating in deep subthreshold are employed. Two different circuit variants describing the neuron model equations have been developed. The first one features spike characteristics, which correlate well with a biological neuron model. The second one is a simplification of the first, designed to exhibit higher spiking frequencies, targeting large scale bio-inspired information processing applications. The most important feature of the fabricated circuits is the energy efficiency of a few femtojoules per spike, which improves prior state-of-the-art by two to three orders of magnitude. This performance is achieved by minimizing two key parameters: the supply voltage and the related membrane capacitance. Meanwhile, the obtained standby power at a resting output does not exceed tens of picowatts. The two variants were sized to 200 and 35 μm^2^ with the latter reaching a spiking output frequency of 26 kHz. This performance level could address various contexts, such as highly integrated neuro-processors for robotics, neuroscience or medical applications.

## Introduction

Computing technology, based on binary coding, Von Neumann architecture and CMOS technology, is currently reaching certain limits (Waldrop, 2016). Traditional computers, the champions for the resolution of complex equation systems, have difficulties to classify/organize data, something that the human brain seems to accomplish effectively. For this reason, research in the field of Artificial Neural Networks (ANNs) is attracting much attention and is quickly developing worldwide. At the bottom of these efforts lies the ultimate goal to realize machines that could surpass the human brain, in some aspects of cognitive intelligence. In that sense, brain research and ANNs bear the promise of a new computing paradigm.

Currently, traditional, discrete-time, digital ANNs, fueled by the unprecedented computational capability of modern Graphics Processing Units (GPUs), represent the state-of-the-art for addressing cognitive tasks (Oh and Jung, 2004; LeCun et al., 2015) such the ones encountered in computer vision applications. However, it is the more recent class of Spiking Neural Networks (SNNs), often referred to as the third generation of neural networks, that are known to be bio-realistic and more computationally potent compared with their predecessors (Maass, 1997). The functional similarity with the actual biological networks permits envisioning, apart from interfacing or reproducing brain processes, the implementation of circuits and systems with cognitive characteristics without explicit programming tasks. This would endow the modern generation of computers with the capacity to learn from input data.

In SNNs, neuronal communication is carried out in the form of noise-robust, signal pulses or “spikes.” SNNs try to reproduce the physical characteristics of the brain, through highly connected neurons dendrites and axons. At present, two main methodologies fulfill neuro-inspired computing tasks: digital simulation and hardware implementation.

In digital simulations, the dynamics of neuronal models are coded in software and calculated on general-purpose digital hardware. Digital simulations have the advantage that they can be reliably programmed using numerical operations of very high precision. However, their reliability comes at the cost of high circuit complexity, which is necessary for the data transfer, exchange and processing (Cao et al., 2015). Accordingly, the energy consumption remains still very high, especially as one juxtaposes biological data for comparison. For instance, the brain of the cat is emulated at the cost of a power dissipation in the megawatt range (Ananthanarayanan et al., 2009), while the animal brain actually consumes only a couple of watts.

As far as hardware implementations of SNNs are concerned, the alternative, “neuromorphic,” approach consists of employing VLSI circuit technology, namely CMOS fabrication processes which can be possibly associated with more advanced device technology such as memristors (Kim et al., 2012). The analog hardware approach consists of a large-scale integration of silicon artificial neurons (AN) and synapses, in an attempt to produce low power neuro-inspired architectures compatible with the current electronics technology.

The efficiency of such architectures can be revealed in contrast to the energy consumption of biological neurons (BN). Brain activity needs a continuous exchange of ions through the cell membrane and these exchanges correspond to the charge and discharge of the neuron capacitance (soma, dendrite, and axon). As a consequence, the important parameters for energy dissipation are the membrane capacitance and the voltage swing. Membrane capacitance varies considerably according to the type of neuron cell, ranging from picofarads to nanofarads for the largest ones (Amzica and Neckelmann, 1999; Golowasch et al., 2009; Rössert et al., 2009; Tripathy and Gerkin, 2012). Interestingly, a recent estimation of the capacitance that could be involved for computation in the human cortex is proposed in Hasler and Marr (2013). The calculations are based on a digital power model and suggest a biological system of 10^12^ neurons with a 0.5 Hz average firing rate. The total capacitance value is calculated at 245 pF that is high when compared with the femtofarad order common in integrated circuits. Indeed, energy savings could be envisioned in silicon AN by aiming at reducing the capacitance and/or the voltage swing.

Next to a reduced capacitance, low power operation in silicon neurons can be facilitated by the physics of the MOS transistors. Indeed, as it has been observed (Mead, 1989, 1990) the nervous system uses, as its basic operation, a current that increases exponentially with the membrane voltage, similar to the current-voltage characteristic of a MOS transistor operating in subthreshold. However, the physical origins of these exponential dependencies are very different: a non-linear voltage controlled conductance in biological membrane against a current controlled by an energy barrier in the transistor. Due to this, the MOS transistor can only asymptotically approach a slope of kT/q per e-fold of current change, while the biology is not limited as such (Mead, 1989, 1990). Even if I-V characteristics show different slopes, a bridge between the physics of biological membrane and the one of electronic devices has been established, especially when the energy and power properties are considered. This led to the advent of neuromorphic silicon neurons, which allowed neuronal spiking dynamics to be directly emulated on analog large-scale integration chips. So far, several generations of SNNs have been proposed and the reader could refer to the relevant works (Misra and Saha, 2010; Indiveri et al., 2011; Hasler and Marr, 2013) to obtain more information.

Based upon these previous works, this paper describes the design and measurement results of a new family of silicon AN. It was designed under the guidelines of (i) a biophysically meaningful model, (ii) a minimum energy dissipation, (iii) an analog circuit that would allow a complete time variation modeling of the membrane potential and (iv) a resulting topology, that when implemented in CMOS technology, it would occupy a minimum area in order to enable large scale integration. This unique combination of characteristics resulted in a neuron topology that was measured to consume several orders of magnitude less energy than the values encountered either for BN or the AN reported so far.

The rest of this paper is organized as follows: The “Materials and methods” section will be devoted to a discussion on neuron energy efficiency, the selection of the mathematical model and the circuit topology and functionality. The circuit proposed in this paper was fabricated and characterized experimentally. Both simulation and experimental results are described in the “Results” section. The “Discussion” section presents a comparison with the state of the art and highlights issues regarding noise, supply voltage sensitivity and temperature impact. Finally conclusions are drawn in the eponymous last section.

## Materials and methods

### Energy efficiency of biological and artificial neurons

In BN, total power dissipation consists of a static and a dynamic part. The major factor of static power dissipation in neuron cells is due to Na-K pumps maintaining a constant resting potential value, despite the sodium and potassium leakage currents. In the circuit design domain, optimizing static power consumption imposes low DC voltage supply and transistors featuring small gate widths.

The dynamic part of energy dissipation is due to the Action Potential (AP) or “spike” generation and propagation. In BN, the AP corresponds to a rapid voltage change between extra and intra-cellular spaces, which are separated by the (quasi-) insulating lipid bilayer. According to the reference model of charge transfer, the two-ion model (Hodgkin and Huxley, 1952), the rising part of the spike is mainly due to an inward sodium current, while the decreasing part is due to an outward potassium current. Changing the voltage across the membrane capacitance *C*_*m*_ by Δ*V*, will need, at least, an amount of charge Δ*Q* = *C*_*m*_Δ*V* to move from one side of the membrane to the other. Since the driving potentials on both sides of the membrane (the Nernst potentials) are constant, the minimum energy dissipated for this change will be Δ*E* = ½*C*_*m*_Δ*V*^2^. If we consider the generation and propagation of a spike (charge and discharge), the total energy dissipated by the cell will be at least *C*_*m*_Δ*V*^2^.

The driving force for the inward and outward currents are the Nernst potentials *E*_*na*_ and *E*_*k*_, which are almost constant in time. In living beings, *E*_*k*_ varies from −80 to −100 mV, while for *E*_*na*_ it is from +30 to +55 mV (Hodgkin and Huxley, 1952; Morris and Lecar, 1981; Wei et al., 2014). The membrane potential always stays between *E*_*k*_ and *E*_*na*_ and *in vivo* measurements showed that the resting potential is close to *E*_*k*_ while the Δ*V* for a spike is close to 100 mV (Hodgkin and Huxley, 1952). Using Δ*V* = 100 mV and *C*_*m*_ = 245pF (Hasler and Marr, 2013) means at least 1.5 × 10^8^ sodium (resp. potassium) ions are entering (resp. leaving) the neuron at each AP and the corresponding energy loss is about *C*_*m*_Δ*V*^2^ = 2.45 pJ.

Starting from these simple considerations, it is clear that decreasing the dissipated energy in AN passes from setting *C*_*m*_ and Δ*V* to the smallest possible values. For artificial neuron circuits, the spike amplitude is proportional or strongly related to the applied DC voltage (Indiveri et al., 2011). Usually, the DC voltage of these circuits is about 1V. Since the energy loss varies with Δ*V*^2^, the smallest possible value for Δ*V* is desired. Meanwhile, maintaining compatibility with the biological neuron spike amplitude, a feature of interest for some applications, entails fixing Δ*V* to about 100 mV. This implies a DC supply voltage of about 150 to 200 mV and constitutes a strong constraint on the active devices comprising the circuit. In particular, the transistors should operate in deep subthreshold regime characterized by a gate voltage far below the threshold, which currently varies between 0.3 to 0.5 V for advanced fabrication nodes. In this regime, however, MOS transistors show some interesting characteristics, such as the exponential control of the drain current by the gate voltage, a low current and therefore low energy dissipation, but they also present some drawbacks, such as a limited transconductance leading to extremely low voltage and power gains.

The other parameter targeted for low energy dissipation is the membrane capacitance. In artificial neuron circuits three main components can be distinguished: the discrete capacitance added in the circuit, and the parasitic capacitance of interconnects. The gate capacitance of transistors was found negligible for the small transistor sizes used in this work. Since the membrane capacitance value has a strong impact on several circuit parameters (energy loss, membrane charging and discharging times, spiking frequency, membrane voltage noise), its value is of utmost importance. For this reason, a mathematical model for the neuron is necessary to provide the necessary guidelines for circuit design.

### Neuron model selection: the Morris-Lecar model

The implementation of an artificial neuron circuit is defined by the selection of a corresponding mathematical model. An analysis of the various advantages and drawbacks of the different mathematical neuron models was proposed in Izhikevich (2004). The Leak Integrate and Fire (LIF) model is one of the simplest and most widely used for such implementations (van Schaik, 2001; Indiveri et al., 2011). However, it is not biophysically realistic: it does not accurately represent the dynamics of ion transport through the neuron membrane. In the analysis of Izhikevich (2004), it was shown that from a biophysical standpoint two models were meaningful: The Hodgkin-Huxley (HH) model (Hodgkin and Huxley, 1952) and the Morris-Lecar (ML) model (Morris and Lecar, 1981). The HH and refined-HH models (Wei et al., 2014) are considered as reference for in depth analysis of the neuron non-linear behavior. Nevertheless, the connection between the HH model and transistor physics is not easily discernible. When transferred to the circuit domain, the use of four non-linear differential equations and multiple non-linear parameters would lead to a relatively complicated topology, demanding considerable silicon surface (Yu and Cauwenberghs, 2010). On the contrary, the ML model is much more attractive for this purpose (Patel and DeWeerth, 1997). It is compiled as a system of two non-linear differential equations associated with exponential functions (Equations 1–5).

(1) $$\begin{array}{l} C_{m}\frac{dV_{m}}{dt}=I_{ex}-G_{Ca}m_{ss}\left(V_{m}\right).\left(V_{m}-E_{Ca}\right)\\ -\;G_{K}.n.\left(V_{m}-E_{K}\right)-\;G_{L}\left(V_{m}-E_{L}\right) \end{array}$$

(2) $$\begin{array}{l} \frac{dn}{dt}=\;\lambda \;\left(V_{m}\right).\;\left(n_{ss}\left(V_{m}\right)-n\right) \end{array}$$

(3) $$\begin{array}{l} m_{ss}\left(V_{m}\right)=\frac{1}{2}.\left[1+Tanh\;(\frac{V_{m}-V_{1}}{V_{2}})\right] \end{array}$$

(4) $$\begin{array}{l} n_{ss}\left(V_{m}\right)=\frac{1}{2}.\left[1+Tanh\;(\frac{V_{m}-V_{3}}{V_{4}})\right] \end{array}$$

(5) $$\begin{array}{l} \lambda \left(V_{m}\right)=\;\lambda_{0}.\text{ Cosh }\left(\frac{V_{m}-V_{3}}{2V_{4}}\right) \end{array}$$

In the above equations the symbols are explained as follows: *C*_*m*_ is the membrane capacitance, *I*_*ex*_ is the excitatory current, *E*_*K*_, *E*_*Ca*_, and *E*_*L*_, are the ion equilibrium potentials, *G*_*Ca*_*, G*_*K*_*, G*_*L*_ are the Ca, K and leak conductances, *n*_*ss*_ and *m*_*ss*_ are the steady-state potassium and sodium gating variables, λ_0_ is the reference frequency and *V*_1−4_ are fitting parameters that can serve for tuning the dynamic properties in order to represent different systems of interest.

The ML model features only two state variables: the membrane voltage *V*_*m*_ and the potassium gating variable *n*. It is well suited to perform phase plane analysis, an efficient tool for analyzing the non-linear dynamics of neurons in terms of stability, spiking conditions and spiking modes (Izhikevich, 2007). Acknowledging this fact, the next section details analytically the design of a circuit response approximating the ML equations. Though exact 1-1 circuit representation of the ML equations is not pursued, this approach yields an energy-efficient design, whose response achieves a good correlation with a higher-order cortical neuron model (Section Biomimetic neuron). As the ML model was initially conceived referring to a barnacle muscle, the following analysis uses Na and K ions (instead of Ca and K) aiming to indicate in the notation the neuronal context, which is ultimately addressed.

### The artificial neuron circuit design

Expressing the system of equations of the ML model as a circuit response can be carried out through a series of considerations for the various components of the system. The most apparent involves the two differential equations to be represented by expressions based on current node summation, as per Kirchhoff's Current Law (KCL). Also, Na and K channel dynamics can be represented by single transistors biased in subthreshold, as it has been previously shown (Farquhar and Hasler, 2005; Hynna and Boahen, 2007).

**Figure 1:**
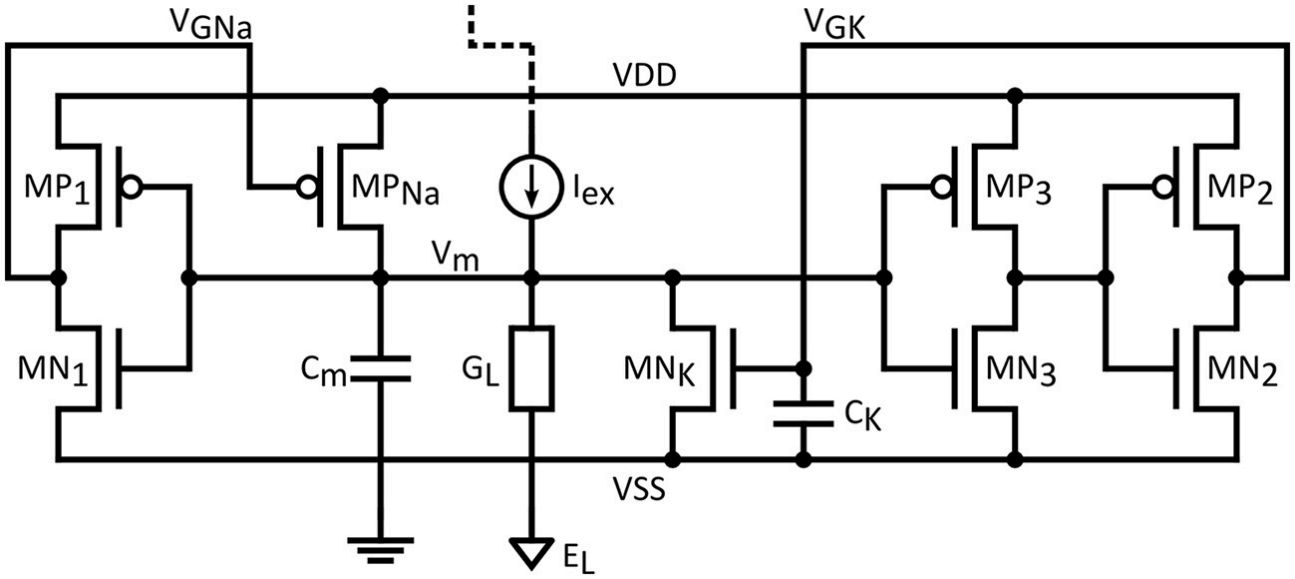
**Proposed biomimetic artificial neuron circuit based on the ML model**.

Figure 1 shows the proposed neuron circuit, based on the ML model described above. The membrane capacitance, *C*_*m*_, is charged through *MP*_*Na*_ (a PMOS transistor modeling the sodium channel) and discharged through *MN*_*K*_ (an NMOS transistor modeling the potassium channel) and the leakage conductance *G*_*L*_. The membrane voltage node is the summing point of the excitation current, leak currents as well as two feedback loops: A positive feedback loop through *MP*_*Na*_ and *MP*_1_/*MN*_1_, which implements a pull-up network for *V*_*m*_, and a negative feedback loop, which implements a pull-down network through *MN*_*K*_ and the two cascaded inverters *MP2*/*MN2* and *MP3*/*MN3*. The time constant of the negative-feedback loop is set by capacitance *C*_*K*_.

The excitation current *I*_*ex*_ is assumed to be provided by a synaptic circuit, which in the most rudimentary form can be implemented by a single transistor current source.

The circuit analysis for the proposed circuit is divided in three parts: the drain current model, the static properties of inverters in subthreshold operation and the circuit response equation.

#### Drain current model in the sub-threshold regime

All the transistors used in the circuit are assumed to operate in the deep subthreshold regime, with a supply voltage lower than 200 mV. The most important effect to be observed from a modeling standpoint is the basic exponential relation for the gate controlled drain current. For this reason, the corresponding expression has been chosen as:

(6) $$\begin{array}{l} \;{\text{I}}_{ds}={\text{G}}_{0}.\exp\left(\frac{V_{gs}}{\eta V_{t}}\right).V_{ds} \end{array}$$

*G*_0_ is the device conductance, that will be referred in the following as *G*_*n*0_ for NMOS and *G*_*p*0_ for PMOS, *V*_*gs*_ and *V*_*ds*_ are the gate-to-source and drain-to-source voltages, η is the subthreshold slope factor and *V*_*t*_ is the thermal voltage. For the sake of simplicity, it is assumed that the NMOS and PMOS ideality factors are the same.

As it will be proved, the use of such a simple model provides a comfortable medium for demonstrating the correlation between the circuit response and the ML model equations sufficiently. In addition to that, it allows a straightforward circuit analysis that is essential for the implementation effort.

#### Static properties of inverters in sub-threshold

**Figure 2:**
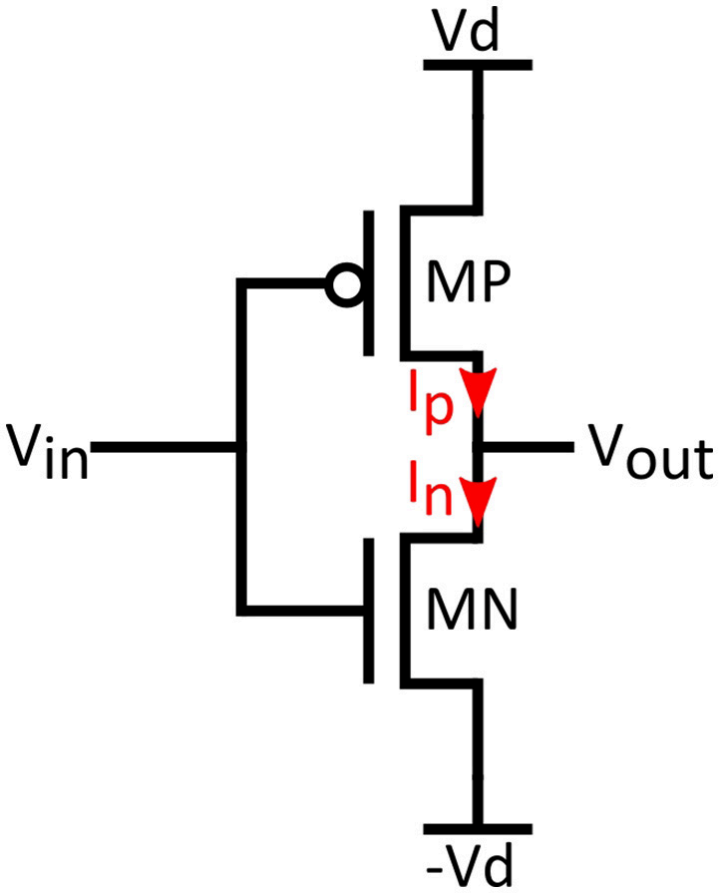
**Inverter biasing conditions used in the analysis**.

The role of inverters is central to circuit performance as the inverter is the means of realizing the necessary Tanh functions in Equations (3,4). We assume a CMOS inverter like the one in Figure 2, operating in the sub-threshold regime. Supply voltages are +/−*V*_*d*_ and input/output voltages are *V*_*in*_ and *V*_*out*_. By applying KCL for the output node of the inverter in Figure 2, and expressing currents as in Equation (6) (assuming same slope factors for NMOS and PMOS), the output voltage can be found as:

(7) $$\begin{array}{l} V_{out}=-V_{d}.Tanh\left[\;\frac{V_{in}}{\;\eta V_{t}}+\frac{1}{2}ln\left(\frac{G_{n0}}{G_{p0}}\right)\right]\\ =-V_{d}.Tanh\left(\frac{V_{in}-V_{isv}}{\;\eta V_{t}}\right)=-V_{d}.Tanh\left(\frac{V_{q}}{\;\eta V_{t}}\right) \end{array}$$

In Equation (7) we simplify the operand of the Tanh function with the use of *V*_*isv*_, that is the input voltage, provided that *V*_*out*_ = 0, then:

(8) $$\begin{array}{l} V_{isv}\equiv {V_{in}|}_{V_{out}\;=\;0}=-\frac{\eta .\;V_{t}}{2}.ln\left(\frac{G_{n0}}{G_{p0}}\right) \end{array}$$

According to Equation (8), *V*_*isv*_ can be either positive or negative depending on the conductance ratio. For further simplification, we define the parameter *V*_*q*_ as:

(9) $$\begin{array}{l} V_{q}=V_{in}-V_{isv} \end{array}$$

The above expression shows two important properties of the proposed circuit. Primarily, the control of the conductance ratio allows adjusting the inverter switching voltage. With a ratio of 10 between the NMOS and PMOS conductance, the switching threshold can be shifted around 50 mV. With this technique, the required voltage shifts V_1_ and V_3_ in Equations (3,4) can be tuned sufficiently. Secondarily, the presence of voltage gain is essential to match the behavior of BN. As it has been shown by Mead (1989), the high sub-threshold slope of MOS transistors (larger than 60 mV/dec) yields a much lower current-voltage slope than the one in biology. So, correct circuit operation is associated with the presence of inverter gain.

In Equation (7), the maximum voltage gain will be obtained for *V*_*out*_ = 0. This can be expressed through differentiation of as:

(10) $$\begin{array}{l} A_{V}={\frac{dV_{out}}{dV_{in}}|}_{V_{in}\;=\;V_{isv}}=-\frac{V_{d}}{\eta V_{t}} \end{array}$$

According to Equation (10), the magnitude of *A*_*v*_ can be larger than one for *V*_*d*_ > η*V*_*t*_. For an η*Vt* about 35 to 40 mV, a significant voltage gain can be provided when *V*_*d*_ > 70–80 mV. It is worth mentioning that this supply voltage value is close to the Nernst potentials encountered in biological cells. This last observation reveals that the neuron circuit can be implemented to operate under extremely low supply bias.

#### The circuit response

**Figure 3:**
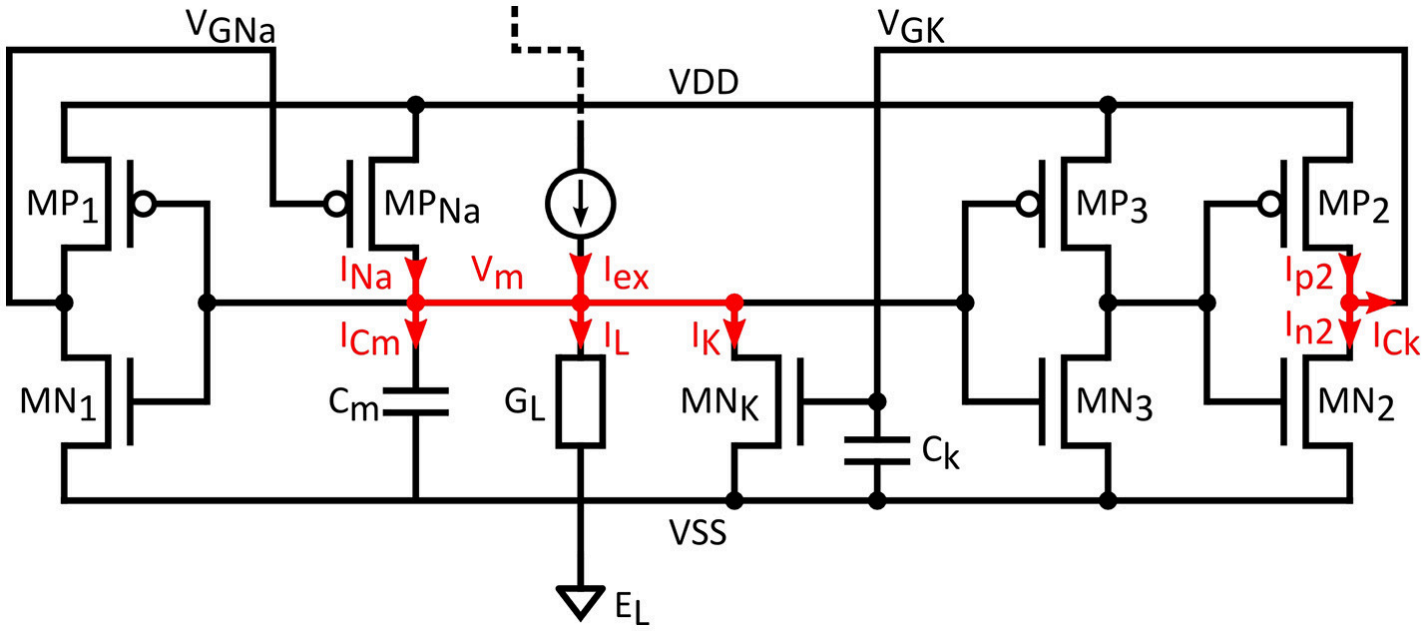
**Artificial neuron circuit analysis**.

To show the connection between the Morris-Lecar equations and the circuit response, we use the circuit shown in Figure 3. In order to simplify the equations and without loss of generality, a symmetrical supply voltage is considered.

Neglecting transistor capacitances and assuming only the capacitances shown in the schematic, two circuit equations are derived from the application of KCL on the two summing nodes *V*_*GK*_ and *V*_*m*_:

(11,12) $$\begin{array}{l} \{\begin{array}{c} I_{C_{m}}=I_{Na}-I_{K}+I_{ex}-I_{L}\\ I_{Ck}=I_{p2}-I_{n2} \end{array} \end{array}$$

In the equations above, *I*_*Na*_ and *I*_*K*_ are the drain currents of MP_*Na*_ and MN_*K*_, *I*_*ex*_ and *I*_*L*_ the excitation and leak currents while *I*_*p*2_ and *I*_*n*2_ are the drain currents in the MP_2_/MN_2_ inverter.

Employing Equation (6) for the drain currents, gives:

(13,14) $$\begin{array}{l} \{\begin{array}{c} \begin{array}{l} C_{m}\frac{dV_{m}}{dt}=G_{Na}.\;exp\left(\frac{V_{d}\;-\;V_{GNa}}{\eta V_{t}}\right).\left(V_{d}-V_{m}\right)\\ -\;G_{K}.\;exp\left(\frac{V_{GK}\;+\;V_{d}}{\eta V_{t}}\right).\;\left(V_{m}+V_{d}\right)+\;I_{ex}-I_{L}\left(V_{m}\right) \end{array}\\ \begin{array}{l} \;C_{k}\frac{dV_{GK}}{dt}=G_{p2}.\exp\left(\frac{V_{d\;}-V_{out3}}{\eta V_{t}}\right).\left(V_{d}-V_{GK}\right)\\ \;-\;G_{n2}.\exp\left(\frac{V_{out3}\;+\;V_{d\;}}{\eta V_{t}}\right).\left(V_{d}+V_{GK}\right) \end{array} \end{array} \end{array}$$

The *V*_*GNa*_ and *V*_*GK*_ are the gate voltages of *MP*_*Na*_ and *MN*_*K*_, and *V*_*out*3_ is the output of *MP*_3_/*MN*_3_ inverter. *V*_*GNa*_ is the output of the MP_1_/MN_1_ inverter and *V*_*out*3_ the output of *MP*_3_/*MN*_3_ both of which are related to *V*_*m*_ by an input/output relation such as Equation (7).

It can be easily proven that the last equation could also be rewritten as:

(15) $$\begin{array}{l} \frac{dV_{GK}}{dt}=\lambda \left(V_{m}\right)\left[V_{d}.\text{Tanh}\left(\frac{V_{q2}}{\eta V_{t}}\right)-V_{GK}\right] \end{array}$$

With:

(16) $$\begin{array}{l} \lambda \left(V_{m}\right)=\frac{2.\text{Cosh}\left(\frac{V_{q2}}{\eta V_{t}}\right).\exp\left(\frac{V_{d}}{\eta V_{t}}\right).\sqrt{G_{n2}.G_{p2}}}{C_{k}} \end{array}$$

and *V*_*q*2_, as seen above:

(17) $$\begin{array}{l} V_{q2}=V_{in2}-V_{isv2}=V_{out3}+\frac{\eta V_{t}}{2}ln\left(\frac{G_{n2}}{G_{p2}}\right) \end{array}$$

The differential equation for *V*_*GK*_ is similar to that of the Morris-Lecar model. In particular, the time dynamics of *V*_*GK*_ are defined by the reference frequency λ*(V*_*m*_*)*, which is related to the circuit parameters through Equation (16).

Introducing the parameters *m*_*ss*_*(V*_*m*_*)* and *n*, Equation (13) could be rewritten as the first differential equation of the Morris-Lecar model:

(18) $$\begin{array}{l} C_{m}\frac{dV_{m}}{dt}=G_{na}.\exp\left(\frac{2V_{d}}{\eta V_{t}}\right).m_{ss}\left(V_{m}\right).\left(V_{d}-V_{m}\right)\\ -G_{k}.\exp\left(\frac{2V_{d}}{\eta V_{t}}\right).n.\left(V_{m}+V_{d}\right)+I_{ex}-\;I_{L}\left(V_{m}\right) \end{array}$$

With:

(19) $$\begin{array}{l} n=\exp\left(\frac{V_{GK}-V_{d}}{\eta V_{t}}\right)\; \end{array}$$

and

(20) $$\begin{array}{l} m_{ss}(V_{m})\;=\;\text{exp}(-\frac{V_{GNa}(V_{m})+V_{d}}{\eta V_{t}}) \end{array}$$

It is easy to see that, *m*_*ss*_*(V*_*m*_*)* and *n* are between 0 and 1 because *V*_*GNa*_ and *V*_*GK*_ are between −*V*_*d*_ and + *V*_*d*_. The *m*_*ss*_*(V*_*m*_*)* function is very well correlated with the Tanh function of the Morris-Lecar model and *n*, the potassium gating variable, is simply related to *V*_*GK*_.

Assuming a small *V*_*GK*_, the first order approximation of *n* would be:

(21) $$\begin{array}{l} n=\exp\left(\;\frac{-V_{d}}{\eta V_{t}}\right)\exp\left(\frac{V_{GK}}{\eta V_{t}}\right)\approx \exp\left(\;\frac{-V_{d}}{\eta V_{t}}\right).\;\left(1+\frac{V_{GK}}{\eta V_{t}}\right) \end{array}$$

This linear relation between *n* and *V*_*GK*_ allows us to rewrite Equation (15) as:

(22) $$\begin{array}{l} \frac{dn}{dt}=\lambda \left(V_{m}\right)\left[n_{ss}\left(V_{m}\right)-n\right] \end{array}$$

which matches the second differential equation (Equation 2) of the Morris-Lecar model.

Equations (13,14) show that the dynamic behavior of the circuit is characterized by two time constants, which are associated with the capacitances *C*_*m*_ and *C*_*k*_ and the currents delivered by the transistors *MP*_*Na*_, *MN*_*K*_, and *MN*_2_/*MP*_2_. The static power of the AN is simply given by the product of the supply voltage (+/−*V*_*d*_) by the leakage currents flowing through the inverters, the leak conductance (if present), and the transistors *MP*_*Na*_ and *MN*_*K*_. The additional energy dissipation during spiking (dynamic power) is related to the charge and discharge of the capacitances *C*_*m*_ and *C*_*k*_.

To conclude this section, it has been formally shown how the proposed circuit response approximates the Morris-Lecar model equations. The main components of this approach have been the use of a simple subthreshold drain current model (Equation 6), the assumption of equal slope factors for PMOS and NMOS and a linearization step. This analytical model was used as a guideline for circuit design and was complemented by more rigorous circuit simulations.

### A simplified neuron circuit

**Figure 4:**
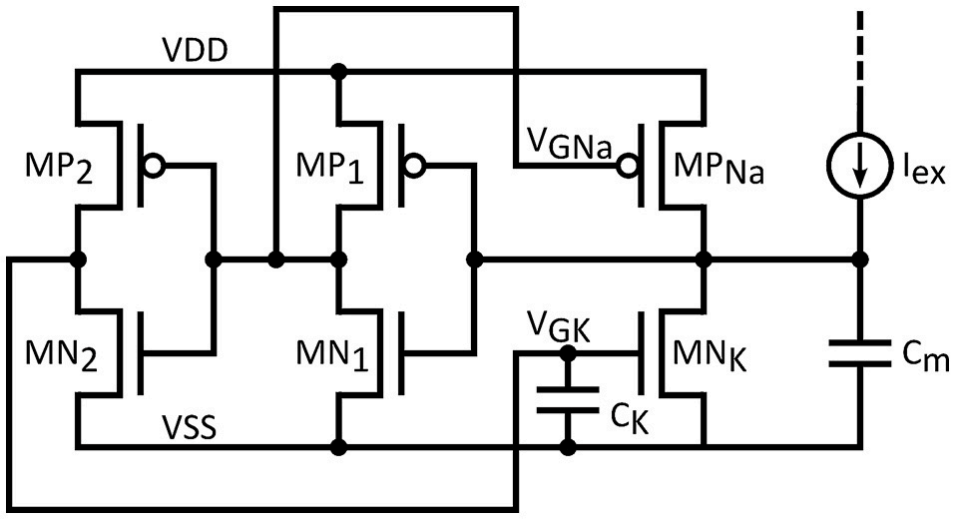
**Simplified neuron circuit**.

If biological accuracy is not of prime concern it could be traded-off for a reduced silicon footprint and a high output frequency performance. Such a modification is suggested in Figure 4. In this circuit, the characteristics of the feedback loops (dynamics, threshold voltage etc.) cannot be set independently, because of the presence of a common inverter stage *MP*_1_/*MN*_1_. Further simplification is obtained by the removal of the leak conductance. The resulting circuit response might not reach the biological relevance of the initial circuit, yet it is still interesting, especially when combined with low capacitance values. Such a lightweight configuration, referred as “simplified neuron” in the following, could adhere to a wider range of frequency specification, aiming at potential deployment in SNNs for computational applications. As it is exhibited below, though the original spike form is not retained and initial design flexibility is compromised, the spiking nature of the output remains intact and maximum output frequency is increased by two orders of magnitude with respect to the biology. Obviously, the approach in circuit analysis seen above is still valid.

### Circuit fabrication and measurement setup

**Figure 5:**
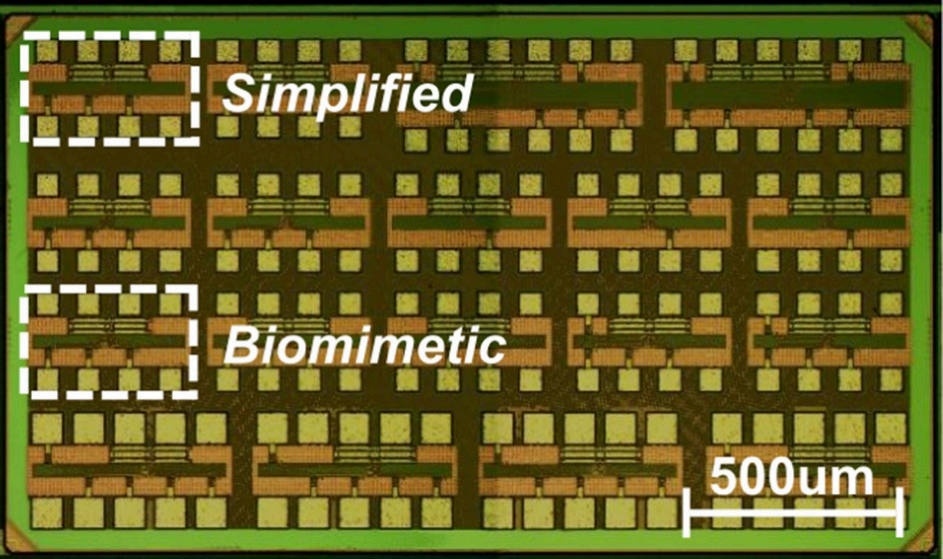
**Die photograph of the fabricated chip**. The highlighted DUTs, each one sized 450 × 250 μm, refer to the two measured neuron circuit variants: biomimetic (core size: 300 μm^2^) and simplified (core size: 35 μm^2^).

The ML neuron circuits described in the preceding sections have been designed and fabricated using TSMC 65 nm CMOS process in the LP option. The test chip size was 1.2 × 2.1 mm and comprised a variety of neuron circuits including variants of the aforementioned biomimetic and simplified neuron topologies. The area occupied by a single neuron circuit ranges from 35 μm^2^ for the simplified neuron to 200 μm^2^ for the biomimetic version without accounting for the output driving buffer or protection circuitry. The capacitors typically dominate the area utilization: 65% for the simplified and 70% in the biomimetic version. A photograph of the fabricated chip is shown in Figure 5. The designs presented here are highlighted.

All the neuron circuits were designed with external biasing. The excitation was implemented with on-chip externally biased transconductance. The output was monitored through an on-chip unity gain output buffer that was designed ensuring that the frequency response of the neuron circuit would not be affected. The output buffer featured independent DC supply to enable accurate power consumption measurements while the neuron's bias pins were not ESD protected. Concerning the results presented in the following section: static power refers to a zero excitation condition, while dynamic power consumption is deduced from the consumption under constant excitation, which induces a spiking output.

The experimental set-up was composed of a waveform generator that generated step signals for the neuron excitation. The supplies used for biasing enabled measurement of the average current with a nominal 500 fA accuracy. The output signal issued from the buffer was monitored by an oscilloscope, in order to perform spike width, frequency and amplitude measurements.

## Results

In this section, we will focus on the performance of the proposed biomimetic and simplified neuron circuits. After describing the basic functionality, the measurement results are presented, regarding spike shape and frequency, standby power and energy dissipation per spike.

### Basic circuit operation

**Figure 6:**
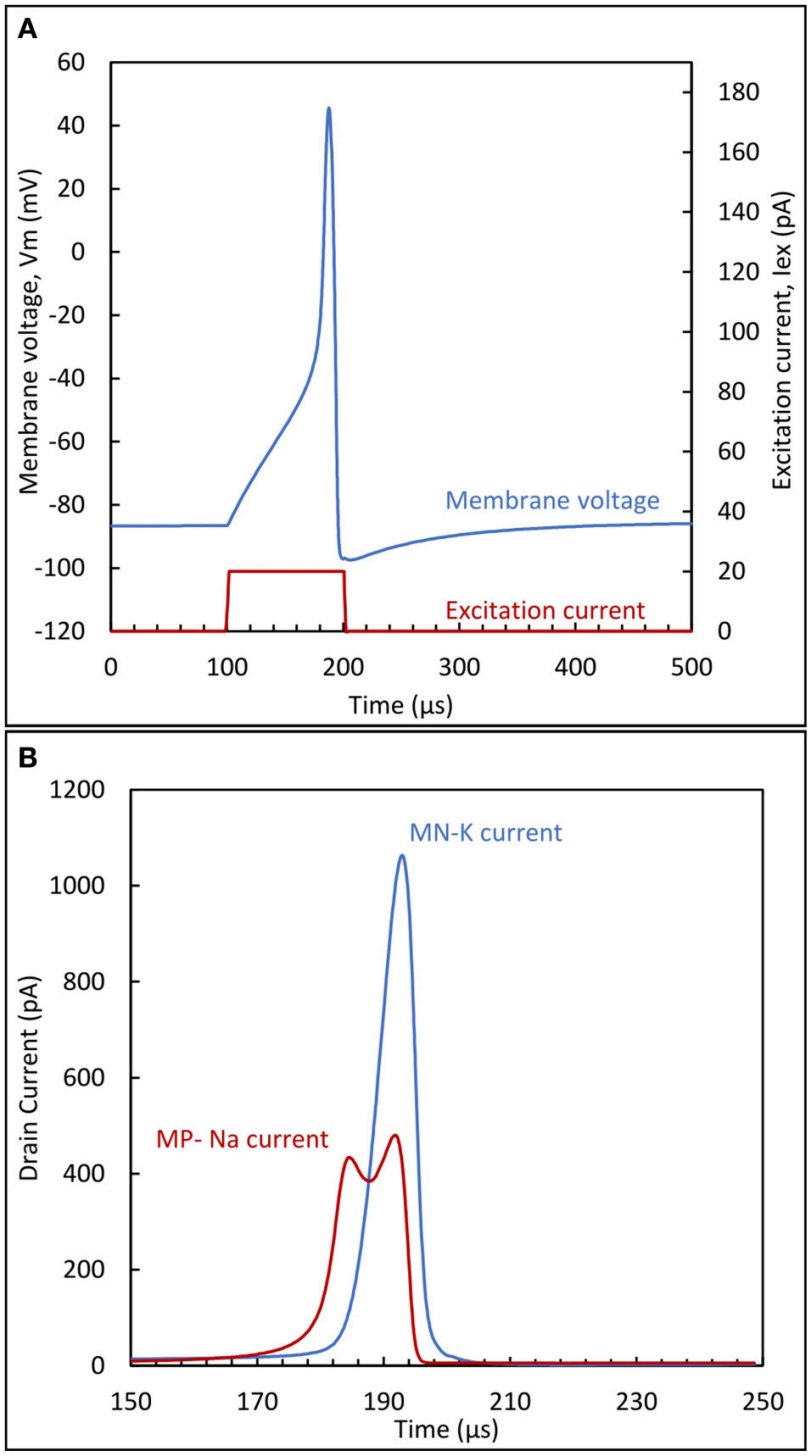
**(A)** Membrane potential and excitation current; **(B)** sodium and potassium currents during the action potential peak. The circuit parameters are: *VDD* = 100 mV, *VSS* = −100 mV, *C*_*m*_ = 20 fF and *C*_*K*_ = 60fF.

To supplement the mathematical analysis above with more intuition, circuit operation can be clarified by following the circuit dynamics for the membrane voltage (Figure 6A) and the drain currents of *MP*_*Na*_ and *MN*_*K*_ transistors (Figure 6B). The following description applies to both presented variants equally.

When excitation is not present, a constant membrane voltage *V*_*m*_ is obtained, assuming a stable circuit. This constant voltage is known in neuron physiology as the resting potential *V*_*rest*_. In BN, this value is close to the potassium Nernst potential. For the circuit, this means that *V*_*rest*_ should be essentially pulled down close to the negative supply. This is achieved by proper sizing of the transistors representing the ion channels. The conductance of *MN*_*K*_ should be much higher than the one of *MP*_*Na*_, in order to achieve a small voltage drop across *MN*_*K*_ source and drain terminals.

While in a rest state, with *V*_*m*_ close to the negative rail, there exists a small current through *MN*_*K*_ and *MP*_*Na*_. Adding an excitation current *I*_*ex*_ will induce further charge accumulation in *C*_*m*_. However, if this current is not sufficient to overcome the current that can be sustained in the *MN*_*K*_ branch, then due to its partial compensation, *V*_*m*_ will be slightly increased on a higher level than the initial resting potential, but still remain constant. On the contrary, if the excitation current exceeds this limit, this will cause *V*_*m*_ to increase with a slope proportional to the excess current: this situation is illustrated in Figure 6A by the quasi-linear increase of membrane potential after 100 μS. If the duration of this excitation is enough to reach the switching threshold of the first inverter (*MN1*/*MP1*), the output will exhibit a steep ascent through the positive feedback action of *MP*_*Na*_. This happens in the vicinity of −40 mV in Figure 6A. This steep increase is due to the exponential relationship between the drain current of *MP*_*Na*_ and its gate voltage, *V*_*GNa*_.

Following the increase, as *V*_*m*_ approaches the positive supply value, the source-drain voltage of *MP*_*Na*_ decreases. This will effectively limit the rise of *V*_*m*_, because the current though *MP*_*Na*_ diminishes as shown in Figure 6B. In the mean-time, another phenomenon takes place. As *V*_*m*_ rises, capacitor *C*_*k*_ is charged and the drain current of transistor *MN*_*K*_ is increased. This action acts antagonistically to the reason creating it, i.e., to the rise of *V*_*m*_, therefore constituting negative feedback. *V*_*m*_ reaches its maximum value when the drain currents of *MP*_*Na*_ and *MN*_*K*_ are equal. After this point the increase in the *MN*_*K*_ drain current will discharge the membrane capacitance, returning *V*_*m*_ eventually back to its resting value or, depending on the excitation, to a value where the current charge accumulation in *C*_*m*_ recommences.

As mentioned before, the above described dynamics and the output spike form rely mainly on circuit characteristics such as the conductance ratio of *MP*_*Na*_ and *MN*_*K*_, the switching threshold of the inverters, as well as the values of the associated capacitances for *C*_*m*_ or *C*_*k*_. For the biomimetic version of the proposed circuit, the facility to independently configure the gains and the time constants for the antagonizing loops ensures tuning of the output form parameters, such as the resting potential, the spike amplitude and width, the spiking frequency and the range of the excitation current. This flexibility permits approximating the behavior of biological neuron models, as it is shown in the following paragraph.

### Biomimetic neuron

For various applications (robotics, medicine…), the neuron circuit dynamics should be compatible with the ones of BN. To account for their different characteristics, a reference model proposed in Wei et al. (2014) was used. This model can be considered as an improved Hodgkin-Huxley model, which unifies neuronal dynamics from spikes to seizures using four state variables, aiming at a better understanding of some pathological states. To achieve a compact circuit and low energy dissipation for the biomimetic circuit variant, capacitance values of *C*_*m*_ = 50 fF and *C*_*K*_ = 100 fF were used with no leak conductance introduced to the circuit. The leak component of the ML equations, however, exists in the form of the parasitic leakage current of the transistor MN_*K*_. As it can be easily observed, an extra component modeling the leak has the same effect as increasing the size of the transistor MN_*K*_ (KCL at Vm node in Figure 3). The absence of an explicit component does not compromise the approximation of the mathematical model, but rather limits the available parametrization options. The “ionic” transistor widths were set as *W*_*Na*_ = 0.6 μm and *W*_*K*_ = 1.83 μm and circuit time constants in the millisecond range were achieved. The rest of the circuit parameters can be found in Table 1.

**Table 1:** **Circuit parameters**.

**Width (m), Capacitance (F)**	**MP1**	**MP2**	**MP3**	**MP_*Na*_**	**MN1**	**MN2**	**MN3**	**MN_*K*_**	***C_*k*_***	***C_*m*_***
Biomimetic	400 n	580 n	120 n	600 n	120 n	120 n	650 n	1.83 u	100 f	50 f
Simplified	300 n	360 n	–	400 n	600 n	120 n	–	1.2 u	8 f	4 f

*Transistor widths and capacitor values used for the two circuit variants presented. Transistor length is L = 65 nm for all cases*.

**Figure 7:**
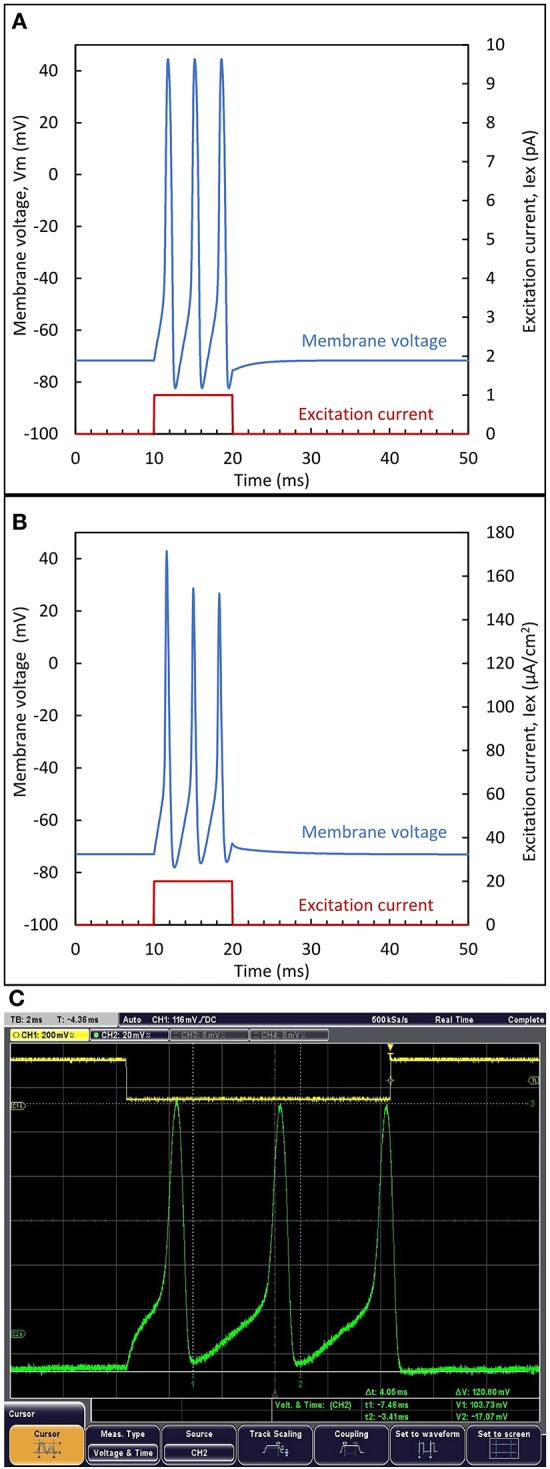
**Biomimetic neuron response to a pulse input current**. Output waveform from **(A)** biomimetic circuit simulation **(B)** reference model and **(C)** experimental response to an excitatory pulse current. The step voltage shown on top, controls the generation of the excitation current.

The comparison between the circuit simulation (A), the reference model (B) and the experiment (C) is illustrated in Figure 7. The Nernst potentials (+55 mV, −92 mV) were applied for circuit biasing, while the reference model plots were created with the code offered with Wei et al. (2014). For the measured results, VSS was set to ground and the supply voltage to 200 mV. The neuron circuit and output was monitored through a buffer. In this comparison, an excitatory pulse current is applied for *Ts* = 10 ms. Since the reference model is based on normalized quantities per unit area (*I*_*ex*_ = 20 μA/cm^2^ and *C*_*m*_ = 1 μF/cm^2^) a common normalization coefficient, ξ = *I*_*ex*_/*C*_*m*_, is used. Figure 7 shows that very similar responses are obtained. For each case, three spikes are generated during the excitation. A slightly decreasing spike amplitude is observed for the reference model, while a constant amplitude is observed for the circuit. The form of the first spike in the measurement differs due to the initial condition imposed by the excitation. Below the supply value of 200 mV, no spikes were observed. This is attributed to the lower transistor currents observed in the experiment, giving rise to a reduced voltage gain (a sufficient voltage gain is required for spiking, as explained in Section Static properties of inverters in subthreshold).

In order to analytically compare the simulation and the experimental results with the reference model, three parameters have been considered: the spiking time *S*_*t*_ (defined as the latency between the maximum of each AP and the beginning of the excitation), the peak to peak amplitude, *V*_*pp*_, and the spike width, *S*_*W*_, defined at *V*_*pp*_/2. These parameters are given in Table 2.

**Table 2:** **Spiking characteristics for a 10 ms excitation current**.

	***S_t_* (ms)**			***V_pp_* (mV)**			***S_w_* (ms)**		
	**Spike 1**	**Spike 2**	**Spike 3**	**Spike 1**	**Spike 2**	**Spike 3**	**Spike 1**	**Spike 2**	**Spike 3**
Reference Model	1.6	5.0	8.3	115.9	101.9	99.7	0.5	0.5	0.5
Circuit Simulation	1.7	5.2	8.6	116.1	116.1	116.0	0.8	0.8	0.8
Measurements	1.8	5.8	9.8	120.8	120.8	120.8	0.6	0.6	0.6

*The values correspond to the three consecutive spikes shown in Figure 7 for each of the three cases*.

In the reference model, *V*_*pp*_ varies from 116 to 99.7 mV while the biomimetic neuron generates spikes with constant peak-to-peak amplitude of 120 mV. This difference is due to the use of the Morris-Lecar model based on two state variables, instead of the four state variables for the Wei model. Nevertheless, a good agreement is observed between the biomimetic neuron circuit and the reference model as far as excitability and spike shape are concerned.

**Figure 8:**
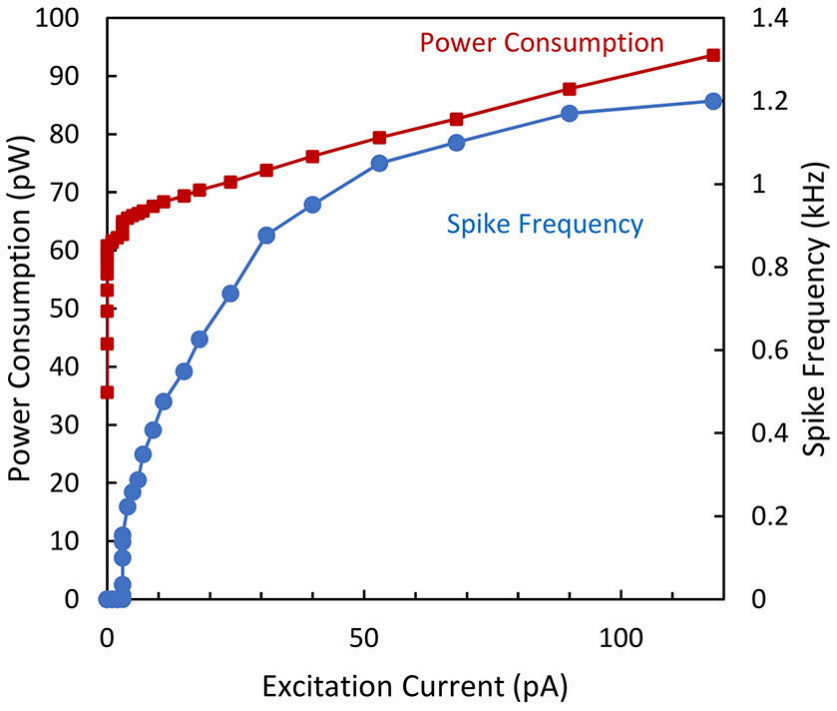
**Spike frequency and power consumption over the excitation current for the biomimetic neuron**.

Figure 8 shows the excitability of the biomimetic neuron. The minimal observed frequency is around 20 Hz. As expected, the firing rate and power consumption increase with the excitatory current. For *I*_*ex*_ = 120 pA, a maximal spike frequency of 1.2 kHz was obtained with a total dissipated power of 90 pW. According to Izhikevich (2007) this biomimetic neuron is categorized as a Type I neuron.

**Figure 9:**
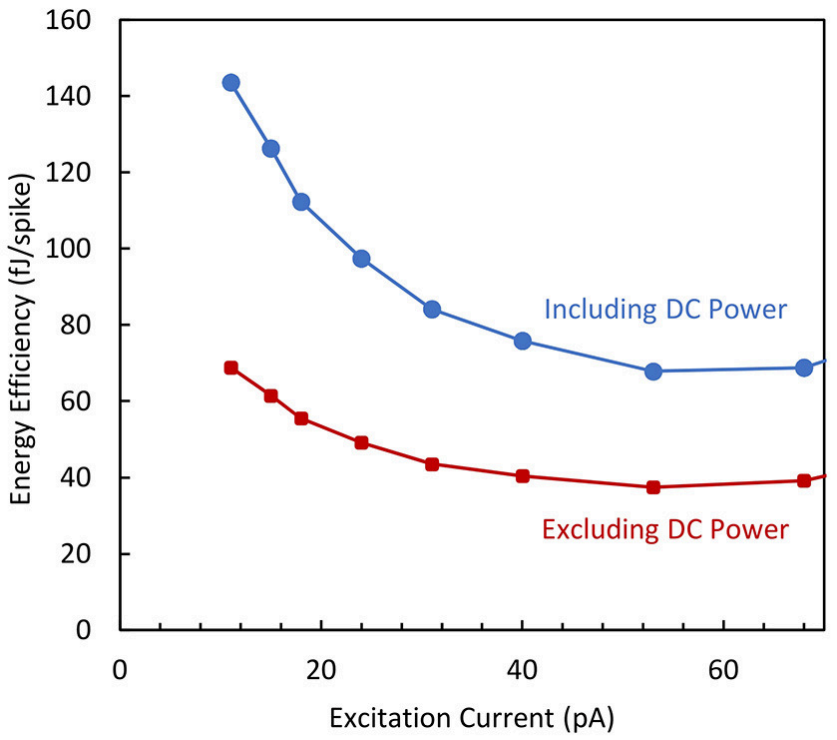
**Energy efficiency vs. excitation current for the biomimetic neuron when the DC power is included or not from energy estimation**.

The energy efficiency of the circuit is deduced from the power dissipation and frequency measurements. In Figure 9 it is plotted as a function of the excitation current for two cases: (i) when the whole average power (static and dynamic) is taken into account and (ii) when the standby power (i.e., power consumption at zero excitation current) is subtracted. For an excitation current higher than 30 pA, the dissipated energy per spike is roughly constant and therefore independent of the spike frequency. An energy efficiency value of 40 fJ/spike is obtained when considering the dynamic power consumption.

It is worth mentioning that this value is several orders of magnitude lower than the energy efficiency of actual BN, as it is estimated in the 10 pJ range, from the ATP consumption in Attwell and Laughlin (2001), Lennie (2003) and Poon and Zhou (2011). This low energy dissipation obtained for the proposed AN can be interesting for SNN applications needing the integration of a large number of neurons.

### Simplified neuron

In this part, the acceleration factor compared to the biological real time (BRT) and the energy dissipation are investigated for the simplified neuron design. Indeed, an important aspect of the simplified neuron, described above, is the ability to operate at accelerated biological time i.e., at higher frequency than the one of the BN. In this context, a circuit was sized to reach the highest spike frequency combined to the lowest energy dissipation. For this purpose, the supply voltage VDD = 200 mV and VSS = 0 V was applied and special emphasis was made to downscale the transistor size: the gate widths were chosen in the sub-micron range (few 100 nm). According to the aforementioned constraints, ultra-low capacitance values were chosen for *C*_*m*_ and *C*_*k*_ values, (4 fF and 8 fF, respectively including parasitic capacitances associated to the interconnections).

**Figure 10:**
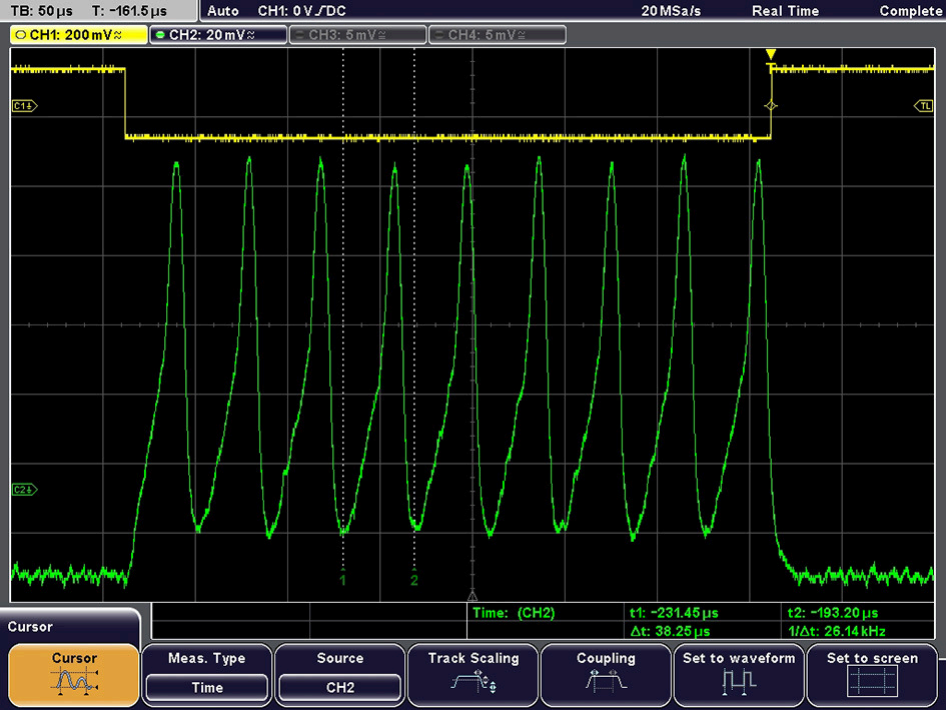
**Example of output waveform under constant current excitation**. Excitation current = 150 pA, Vpeak-peak = 112 mV, Spike frequency = 26 kHz.

The output waveform resulting from an excitatory pulse current is presented in Figure 10, demonstrating the ability of this neuron to be externally stimulated.

**Figure 11:**
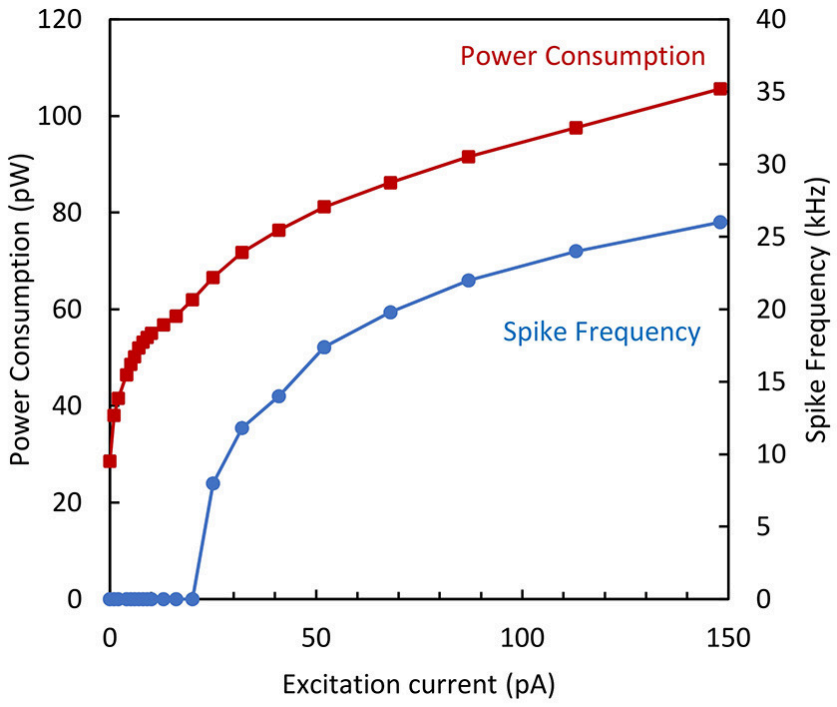
**Spike frequency and power consumption vs. excitation current for the simplified neuron variant**.

In order to analyze the AN performance, both the spike frequency and the corresponding average power consumption (including the static and dynamic parts) have been measured as a function of the excitation current. For the maximum excitation current, a spike frequency as high as 26 kHz was obtained with a total power consumption of 105 pW. The plot of power consumption and spike frequency is shown in Figure 11 as a function of the excitation current.

As already discussed, the main objective of this work was to minimize the energy dissipation: a value is in the fJ/spike range was predicted by the circuit simulation. The energy efficiency was determined from measurements of the total dissipated power along with the spike frequency.

**Figure 12:**
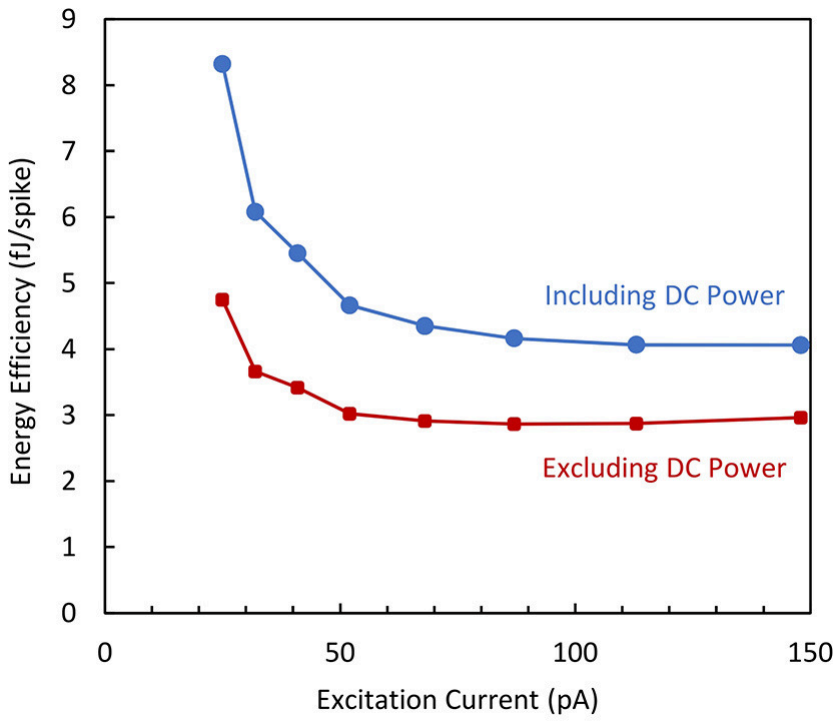
**Energy efficiency vs. excitation current measured for the simplified neuron variant**.

The energy efficiency is plotted as a function of the excitation current in Figure 12 in two cases as previously mentioned for the biomimetic variant of the circuit. The curves demonstrate that the energy efficiency does not significantly depend on the output spiking frequency and the experimental dissipation reaches values as low as 3 fJ/spike when only the dynamic power is considered.

## Discussion

The most important feature of the proposed neuron circuit is the ultra-low energy consumption. This is mainly attributed to the operation under a minimal supply voltage, which entails low drain to source voltage and deep subthreshold operation. Reduced to one sixth of the standard value for this technology node, it actually approaches the membrane potential values met in biology. Meanwhile, the design achieves the desired characteristics with minimum explicit capacitance load. This combination leads to a dynamic power consumption of a few femtojoules per spike for both circuit variants and specifically as low as 4fJ/spike for the simplified neuron. The characterization described in the previous sections refers to neuron circuits that were realized as stand-alone. Performance is benchmarked against similar implementations in Table 3. The values reported for the power consumption include both static (DC) and dynamic (AC) power; hence, the energy efficiency is calculated accounting for the total dissipated power. An improvement of two orders of magnitude below the state of the art energy efficiency in Cruz-Albrecht et al. (2012) is observed, along with a silicon area decrease by more than one order of magnitude. This is a very encouraging result in the road to diminish the power consumption of SNNs. Following, we shortly discuss the effects of noise, temperature and supply voltage variation as well as relevant synapse technology.

**Table 3:** **State of the art performance of reported stand-alone artificial neurons**.

**References**	**Neuron type**	**CMOS node**	**Core area (μm^2^)**	**“Membrane capacitor” value (fF)**	**Spike voltage swing (mV)**	**Spiking frequency (Hz)**	**Power**	**Energy efficiency (/spike)**
Indiveri et al., 2006	LIF^*^	0.35 μm	2,573	432	1,500	200	–	900 pJ
Wijekoon and Dudek, 2008	LIF	0.35 μm	2,800	100	3,000	10^6^	8–40 μW	8.5–9 pJ
Basu and Hasler, 2010	“Saddle”	0.35 μm	2,740	–	150	100	1.74 nW	17.4 pJ
Joubert et al., 2012	LIF	65 nm	538	500	800^†^	1.9 × 10^6^	78 μW	41 pJ
Cruz-Albrecht et al., 2012	LIF	90 nm	442	–	600^†^	100	40 pW	0.4 pJ
This work: Biomimetic	ML-based	65 nm	200	50	120	1.2 × 10^3^^**^	94 pW	78.3 fJ
This work: Simplified	ML-based	65 nm	35	4	112	25 × 10^3^^**^	100 pW	4 fJ

* *LIF:Leaky Integrate and Fire*.

** *Maximum frequency measurement*.

† *Simulation result*.

Firstly, despite the quite low peak to peak spike amplitude (~ 100 mV), the noise, while being observable in Figures 7C, 10 did not seem to strongly perturb the output. If we consider an equivalent circuit for output stage of the neuron circuit composed of a conductance *G*_*m*_ and a capacitance *C*_*m*_ in parallel, the Root Mean Square (RMS) of the generated noise voltage is:

(23) $$\begin{array}{l} v_{m}=\sqrt{\frac{kT_{n}}{C_{m}}} \end{array}$$

where *T*_*n*_ is the noise temperature of the conductance *G*_*m*_ and *k* the Boltzmann constant. Evidently, in contrast to energy dissipation, the noise voltage increases when the membrane capacitance decreases. This fact can pose an issue in performance for a low capacitance circuit. Biased under threshold, the transistor drain current is low and the main noise component is thermal. For an order of a few femtofarads (as in the simplified neuron design), a pure thermal noise (*T*_*n*_ = 300 K) will induce an RMS noise voltage around 1 mV. Such a value was confirmed experimentally, and the first conclusion is that the noise has little impact. It is also worth mentioning that in biology, RMS voltage fluctuations are also in the mV range (Rudolph and Destexhe, 2004) but these fluctuations are obtained for a much higher capacitance (several 10's of pF, see the discussion in the introduction of this paper). This could be explained by the fact that the noise temperature *T*_*n*_ in biology is higher than the one considered for the neuron circuit.

Secondly, considering the behavior of the neuron circuit with the variation of ambient temperature. The use of subthreshold operation denotes an exponential variation of the drain current versus temperature. For SNN applications, the stability of the resting potential versus temperature is essential. For that matter, let us consider the circuit shown in Figure 4, with no excitatory current. In steady state, (assuming VSS set to 0 V), *MP*_*Na*_ and *MN*_*K*_ are off and the resting membrane potential *V*_*rest*_ can be calculated as:

(24) $$\begin{array}{l} V_{rest}=\;\frac{G_{Na}}{G_{Na}+G_{K}}.\;VDD=\;\frac{1}{1+\frac{G_{K}}{G_{Na}}}\;.\;VDD \end{array}$$

By carefully looking at Equation (24), the effect of temperature variation mainly depends upon the ratio between the potassium and sodium conductance. Assuming roughly the same impact of temperature for each transistor (Alioto, 2010), a smooth variation of the membrane resting potential is expected.

**Figure 13:**
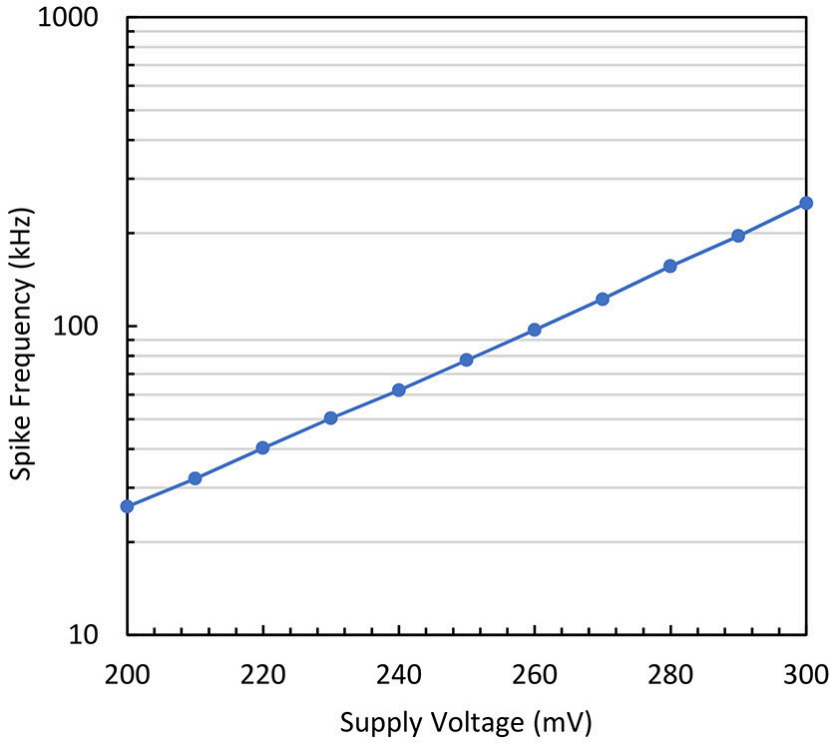
**Output spike frequency measurement vs. supply voltage variation for the simplified neuron variant**.

Thirdly, the impact of the supply voltage variation is investigated. As clearly indicated in Equation (18), the spike frequency is proportional to $\exp\left(\frac{2V_{d}}{\eta V_{t}}\right)=\exp\;\left(\frac{VDD-VSS}{\eta V_{t}}\right)$. Figure 13 shows the measured results of the output spike frequency as the supply voltage (VDD) is increased. A straight line is obtained for the logarithmic plot that demonstrates the exponential relationship that exists between the output time constant and the supply voltage. It is also worth mentioning that the acquired slope of 100 mV/dec is very close to the sub-threshold slope of the transistor. This plot is a good indication of the validity of the analytical model presented above.

**Figure 14:**
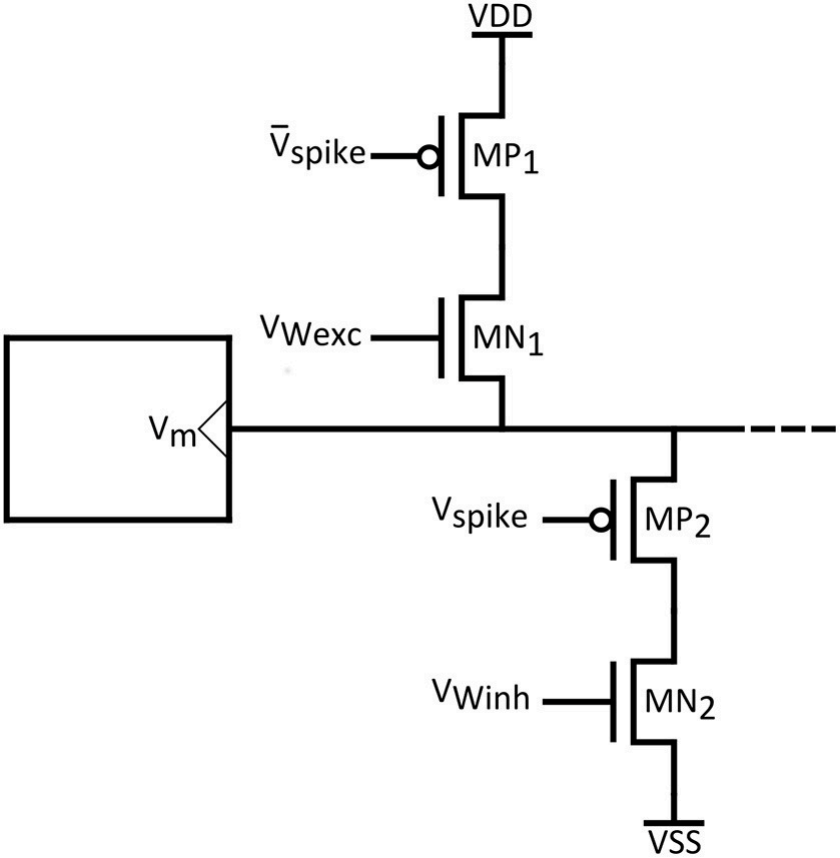
**Indicative synaptic circuit**.

Finally, we would like to emphasize the challenge related to compatible synapse technology. In Figure 14 an indicative synaptic circuit is shown, that can be used to connect the proposed neuron circuits. PMOS transistors can model excitatory synapses through additional sodium channels, while conversely inhibitory synapses would be represented by additional potassium channels and NMOS transistors. As widely supported by many works (Arthur and Boahen, 2006), in order to introduce plasticity, the excitatory and inhibitory post synaptic currents can be controlled by interposing “weight” transistors. In the context of synaptic plasticity, SNNs can feature unsupervised learning through the so-called “Spike Timing Dependent Plasticity” (STDP) rule (Bi and Poo, 1998; Markram et al., 2012). Though use of emergent technologies such as memristors to realize plastic synapses featuring STDP is promising (Yang et al., 2013; Prezioso et al., 2015), issues as cost, reliability and large volume production advocate for the well-established CMOS technology. The STDP scheme proposed in Arthur and Boahen (2006), is fully compatible with our artificial neuron technology and can be readily employed.

## Conclusion

In conclusion, this work presented the properties and experimental results of an artificial neuron circuit based on the Morris-Lecar model that was fabricated in 65 nm standard CMOS technology. Circuit design was inspired from the ML neuron model equations and an analysis is offered in order to formally demonstrate the analogy between the mathematical model and the circuit response. The design of the artificial neuron has been conceived to perform closely to the biological neuron and allow high-level integration. From a circuit design perspective, the choice of using transistors in deep subthreshold operation exposed three key points: (i) the exponential control of the drain current by the gate voltage, (ii) the possibility to easily realize the required non-linear functions (Tanh) by using inverters, and (iii) the operation under minimal supply voltage and related capacitance, which enabled important energy consumption savings. The circuit analysis, through closed form equations, points out several important characteristics. Primarily, achieving voltage gain is essential to match the high slope of current change met in biology. Secondly, the output frequency is expressed as a function of key circuit parameters. Though the ML model is well suited to produce an architecture having a biological relevance, it soon became clear that the resulting topology can be extended to various performance optimization goals. For instance, the design could be simplified to achieve further reduction in power consumption and silicon area. The measured results confirm the systematic approach and the orders of magnitude improvement in energy efficiency over prior state of the art motivate the use of the proposed circuit in energy-efficient spiking neural network architectures.

## Author contributions

AC initiated and supervised this study. VH, SH, FD, CL performed the circuit simulations and measurements. IS designed the chip and EM managed the tape-out submission. All authors discussed the results and contributed to the refinement of the manuscript. IS finalized the manuscript.

### Conflict of interest statement

The authors declare that the research was conducted in the absence of any commercial or financial relationships that could be construed as a potential conflict of interest.
